# Chicago Medical Society

**Published:** 1860-12

**Authors:** 


### CHICAGO MEDICAL SOCIETY.

The regular monthly meeting of this society was held Nov. 16th, the President, Dr. Orrin Smith, in the chair.

After the usual preliminary business, Dr. Ira Hatch read the report of a case of spontaneous inversion of the uterus, and its subsequent history. The report, in full, will be found in the present number of the *Examiner.* An interesting discussion followed, in reference to the efficient cause sof uterine inversion, during which Dr. Wickersham called the attention of the society to a statistical article in the October number of the *American Journal of Medical Sciences*, by Dr. Charles A. Lee. He stated that some of the cases quoted by Dr. Lee, were either quoted erroneously or purposely garbled; and that the conclusions drawn were still more erroneous. All these misstatements and errors very singularly tended to magnify the importance of *traction on the cord*, as the cause of inversion. In view of the importance of the subject, and the direct bearing of such statistics on the reputation of the physician, in whose hands a case of inversion might occur, the society appointed a committee consisting of Drs. Orrin Smith, Ira Hatch, and W. H. Byford, to critically examine the article of Dr. Lee, and expose whatever important errors might be found therein.

The special topic selected for discussion at this meeting was *Scrofula.* The subject was discussed at considerable length by Drs. Waite, Bevan and Hatch. Dr. Waite, among other remedies, recommended the Stillingia as the best vegetable alterative we possess for the treatment of scrofulous diseases. Dr. Bevan raised the question whether there was any necessary connection between syphilis and scrofula, and adduced some facts that would countenance such connection; but not sufficient to demonstrate its actual existence. Dr. Hatch strongly recommended quinine in the treatment of scrofulous opthalmia. The latter disease was selected as the subject for discussion at the next meeting. Dr. N. S. Davis was appointed to read an essay.

The Society then adjourned.

